# Repeated Transcranial Magnetic Stimulation for Improving Cognition in Patients With Alzheimer Disease: Protocol for a Randomized, Double-Blind, Placebo-Controlled Trial

**DOI:** 10.2196/25144

**Published:** 2021-01-08

**Authors:** Zahra Moussavi, Grant Rutherford, Brian Lithgow, Colleen Millikin, Mandana Modirrousta, Behzad Mansouri, Xikui Wang, Craig Omelan, Lesley Fellows, Paul Fitzgerald, Lisa Koski

**Affiliations:** 1 Biomedical Engineering Program The University of Manitoba Winnipeg, MB Canada; 2 Department of Clinical Health Psychology Max Rady College of Medicine The University of Manitoba Winnipeg, MB Canada; 3 Department of Psychiatry Max Rady College of Medicine The University of Manitoba Winnipeg, MB Canada; 4 Vision and Concussion-iScope Clinic Winnipeg, MB Canada; 5 Warren Centre for Actuarial Studies and Research I H Asper School of Business The University of Manitoba Winnipeg, MB Canada; 6 Department of Neurology and Neurosurgery McGill University Montreal, QC Canada; 7 Department of Psychiatry Monash University Melbourne Australia; 8 Department of Psychology Faculty of Science McGill University Montreal, QC Canada

**Keywords:** repetitive transcranial magnetic stimulation, Alzheimer disease, double blind, treatment, placebo controlled, randomized

## Abstract

**Background:**

Alzheimer disease has no known cure. As existing pharmacologic interventions only modestly slow cognitive decline, there is a need for new treatments. Recent trials of repetitive transcranial magnetic stimulation (rTMS) have reported encouraging results for improving or stabilizing cognition in patients diagnosed with Alzheimer dementia. However, owing to small samples and lack of a well-controlled double-blind design, the results to date are inconclusive. This paper presents the protocol for a large placebo-controlled double-blind study designed with sufficient statistical rigor to measure the efficacy of rTMS treatment in patients with Alzheimer dementia.

**Objective:**

The objectives are to (1) recruit and enroll up to 200 eligible participants, (2) estimate the difference in treatment effects between active treatment and sham treatment, (3) estimate the difference in treatment effects between two doses of rTMS applications, (4) estimate the duration of treatment effects among responders to active rTMS treatment, and (5) estimate the effect of dementia severity on treatment outcomes among patients receiving active rTMS treatment.

**Methods:**

We have designed our study to be a double-blind, randomized, placebo-controlled clinical trial investigating the short- and long-term (up to 6 months) benefits of active rTMS treatment at two doses (10 sessions over 2 weeks and 20 sessions over 4 weeks) compared with sham rTMS treatment. The study will include patients aged ≥55 years who are diagnosed with Alzheimer disease at an early to moderate stage and have no history of seizures and no major depression. The primary outcome measure is the change in the Alzheimer Disease Assessment Scale-Cognitive Subscale score from pretreatment to posttreatment. Secondary outcomes are changes in performance on tests of frontal lobe functioning (Stroop test and verbal fluency), changes in neuropsychiatric symptoms (Neuropsychiatric Inventory Questionnaire), and changes in activities of daily living (Alzheimer Disease Co-operative Study-Activities of Daily Living Inventory). Tolerability of the intervention will be assessed using a modification of the Treatment Satisfaction Questionnaire for Medication. We assess participants at baseline and 3, 5, 8, 16, and 24 weeks after the intervention.

**Results:**

As of November 1, 2020, we have screened 523 individuals, out of which 133 were eligible and have been enrolled. Out of the 133 individuals, 104 have completed the study. Moreover, as of November 1, 2020, there has been no serious adverse event. We anticipate that rTMS will considerably improve cognitive function, with effects lasting up to 3 months. Moreover, we expect rTMS to be a well-tolerated treatment with no serious side effect.

**Conclusions:**

This protocol design will allow to address both the rTMS active treatment dose and its short- and long-term effects compared with sham treatment in large samples.

**Trial Registration:**

ClinicalTrials.gov NCT02908815; https://clinicaltrials.gov/ct2/show/NCT02908815

**International Registered Report Identifier (IRRID):**

DERR1-10.2196/25144

## Introduction

### Background

Dementia is a growing problem in our society as life expectancy increases. The leading cause of dementia, Alzheimer disease, has no cure, with current treatment options limited to slowing the progression of cognitive impairment. Recent small-scale clinical trials of high-frequency repetitive transcranial magnetic stimulation (rTMS) have shown some improvement in the cognitive abilities of patients with mild to moderate Alzheimer disease [1-15], with effects that diminish over a period of 2 to 3 months [8].

Cholinesterase inhibitors are the current treatment mainstay for Alzheimer disease. These medications increase the excitability of cells that respond to acetylcholine. The most commonly used medication, donepezil, shows some benefits in 20% to 60% of patients [16], but a substantial and marked benefit in only 2.3% of patients [17]. A long-term follow-up study of donepezil showed no significant benefit compared with placebo for improving or preventing declines in activities of daily living among patients with Alzheimer disease [18]. Moreover, some patients discontinue these drugs owing to severe side effects [16,17]. Thus, better treatments are needed. A few recent studies have suggested that modulating cortical excitability through noninvasive brain stimulation using rTMS is a promising approach to treatment, either alone or in addition to cholinesterase inhibitors [1-15].

rTMS is a noninvasive nonpharmacological technique that is quick to administer and relatively easy for patients to tolerate, with no lasting side effects. It is a procedure in which a series of electric currents are pulsed through a coil placed on the scalp; they produce a time-varying magnetic field [19] that passes through the skull to the brain, wherein a small current is induced in the underlying cortical tissue. Low-frequency pulses (<5 Hz) seem to decrease cortical excitability through the well-described process of long-term depression, while high-frequency pulses (10-20 Hz) seem to increase cortical excitability and synaptic plasticity through long-term potentiation mechanisms [20-22]. Long-term potentiation has a role in synaptic plasticity and is regarded as one of the central cellular mechanisms of learning and memory [23]. rTMS at either low or high frequency has been studied as a potential treatment for a wide variety of neurological and neurodegenerative disorders (eg, depression, Alzheimer disease, Parkinson disease, and stroke). Currently, the use of rTMS is only approved for the treatment of major depressive disorders.

### Development of the Protocol

A few studies [1-8], including some from our team, have explored the possibility of rTMS as a treatment for Alzheimer disease in small samples (<45) with similar protocols, that is, high-frequency rTMS applied bilaterally to the dorsolateral prefrontal cortex (DLPFC). The assessments used in these studies mostly included Mini-Mental State Examination (MMSE) and Alzheimer Disease Assessment Scale-Cognitive Subscale (ADAS-Cog; some only used performance on an object/action naming task). They all reported some improvements over the course of treatment. Not every study had a sham-treated control group. Only one of these studies investigated the durability (up to 3 months) of the treatment among responders. None of these studies reported any adverse effect of the treatment.

To date, such studies have yielded conflicting results, which may be due in part to methodological limitations, such as a small sample size and the lack of a well placebo-controlled double-blind design. We aim to address these limitations in a large sample clinical trial investigating the efficacy of rTMS treatment and the duration of its effects. We will also explore the characteristics of responders and nonresponders.

Previous studies of rTMS in patients with Alzheimer disease used different rTMS protocols (ie, different areas of stimulation, duration and frequency of stimulation, coil type, number of pulses, and intertrain interval), without directly addressing their suitability for Alzheimer disease. The most important parameters of rTMS treatment are stimulation frequency and the location targeted by this stimulation. Many previous studies of rTMS treatment in the context of Alzheimer disease have used high-frequency (10-20 Hz) stimulation bilaterally to the DLPFC in order to increase cortical excitability. The DLPFC plays an important role in executive functions, such as planning, organization, and decision-making, with a well-established key role in working memory. Dementia commonly affects these cognitive processes, and it is related in part to DLPFC dysfunction [24]. Other studies have stimulated the Broca area, Wernicke area, parietal somatosensory association cortex, right frontal gyrus, right superior temporal gyrus, parietal P3/P4, posterior temporal T5/T6, and precuneus, and combinations thereof with varying degrees of success [9-15]. The only large study so far used neuroAD therapy applied over 6 weeks to six sites (three at a time and alternating across days). This study claimed a considerable improvement (ie, 31.7% had a 0-4 ADAS-Cog improvement for active treatment compared with 15.4% for sham treatment) in people with Alzheimer disease and baseline Montreal Cognitive Assessment (MoCA) scores less than 30 [14]. Weiler et al have reviewed the extant literature up to 2018 [25].

Individuals with Alzheimer disease may have profound impairment of metabolic interactions between neurons and astrocytes owing to an abnormal glutamate-glutamine (Glx) cycle [26]. Application of high-frequency rTMS to the left DLPFC area has been shown to increase Glx levels and normalize the Glx cycle [27]. High-frequency rTMS also increases cerebral blood flow and glucose metabolism in stimulated and remote brain regions [28], as well as reduces intracortical inhibition at the stimulation site [29]. High-frequency rTMS applied to the right DLPFC area has been shown to alleviate anxiety symptoms [30], which are considerably higher in patients with Alzheimer disease at mild to moderate stages than age-matched healthy controls [27,28,31,32]. Enhanced synaptic plasticity has been suggested as a potential mechanism for the effects of high-frequency rTMS [21].

Cortical excitability is observed following repetitive high-frequency stimulation [33,34]. Long-term potentiation–like changes in synaptic strength are widely presumed to be a mechanism of learning and memory. It has been shown that 100-Hz magnetic stimulation induces long-term potentiation effects in rat hippocampal slices [35], while related synaptic enhancement has been reported in cortical structures following 10 to 20-Hz stimulation [36,37]. High-frequency rTMS can considerably upregulate brain-derived neurotrophic factor (BDNF) levels [38], which decline within the hippocampus in patients with Alzheimer disease [39]. BDNF levels are affected by neuronal activity and long-term potentiation, which regulate these plasticity-related neurotrophins. Moreover, rTMS is a modifier of inhibitory neuron function. In hippocampal slices, 10-Hz stimulation reduces gamma aminobutyric acid (GABA)ergic synaptic strength in principal neurons. This supports models and mechanisms involving GABAergic synapses modulating the overall inhibitory/excitatory balance [40].

Building on this promising body of research, we chose to apply high-frequency rTMS bilaterally to the DLPFC. We speculated that this would benefit people with Alzheimer disease via enhanced blood flow and glucose metabolism, synaptic plasticity, and improved connectivity. Either side of the DLPFC will likely activate the basal forebrain cholinergic complex, which has projections over most of the cortex and has connectivity via GABAergic inputs to the midbrain regions. New studies have shown a link between GABAergic dysfunction and cognitive function [41-43]. Consequently, the increased excitability of these less GABA-suppressed areas in the brain of patients with Alzheimer disease may allow for increased response in not only cortical regions but also midbrain regions, which are important as major sources of cholinergic, serotonergic, and norepinephrinergic inputs to many regions of the brain.

The other parameters of rTMS treatment selected for this clinical trial, such as the number of pulses, intertrain intervals, and the duration of treatment, are rather arbitrary within a range [44]. They were selected from among specific parameters with demonstrated effectiveness in the extensive rTMS literature on Alzheimer disease [1-15], and depression as a depressive symptom is often comorbid with Alzheimer disease [45].

The intertrain interval in this trial was selected for its efficiency and safety profile as documented in international guidelines for the use of high-frequency rTMS [46,47]. Alternative stimulation protocols, such as theta-burst stimulation [44], are more efficient, but were not selected for study because there is only limited evidence for their effectiveness in Alzheimer dementia.

As for the choice of coil, there are only a few options. Double cone and H coils are used for reaching deep areas of the brain (up to 5-cm penetration), but they have not been used in Alzheimer treatment studies owing to the uncomfortable facial twitches that they may cause during high-frequency stimulation. As nearly all the studies cited herein use the figure-8 coil, this configuration was chosen. Pulses of both figure-8 and round coils penetrate only to the neocortex close to the skull surface of the brain.

### Choosing rTMS Parameters

The investigation of rTMS as a potential treatment for Alzheimer disease presents many challenges. Among these challenges is the multitude of parameters that may impact the efficacy of treatment, including, but not limited to, (1) the target area of stimulation, (2) the total number of pulses, which is also correlated with the duration of the treatment, (3) the frequency of the pulses, (4) the intensity of the pulses (percentage of the resting motor threshold [RMT]), and (5) the protocol of delivery of the pulses (train length, intertrain interval, etc).

Guerra et al considers managing the many variabilities in noninvasive brain stimulation studies [44]. To date, there is no study that can provide convincing answers as to what the optimum parameters are. For a tabularized review on the used rTMS parameters, please refer to a previous report [25]. Thus, we are still at the stage of pilot studies to determine an optimum protocol for Alzheimer rTMS treatment. One main constraint is the number of eligible study participants. This limits the number of protocols to be tested if we desire to have a high statistical power in our outcome measures. This study has endeavored to select reasonable and justifiable values for each of these parameters, mainly based on our previous pilot studies [8,48], which were themselves based on previous work in the field.

### Study Goal, Objectives, and Hypotheses

The overall goal of this study is to compare the efficacy of high-frequency active versus placebo rTMS for the treatment of cognitive impairment among people with mild to moderate Alzheimer dementia. The specific objectives and hypotheses of the study are as presented below.

First, estimate the difference in treatment effects among patients treated with active as compared with placebo high-frequency rTMS applied bilaterally to the DLPFC. Hypothesis 1 (H1) is as follows: better cognitive performance will be seen in patients randomly assigned to the active treatment group compared with those assigned to the placebo group.

Second, estimate the difference in treatment effects for patients receiving 4 weeks of rTMS versus 2 weeks of rTMS. Hypothesis 2 (H2) is as follows: four weeks of rTMS will be more effective than 2 weeks of rTMS in improving cognitive function.

Third, estimate the duration of treatment effects among responders to active rTMS, where response is defined as improvement in the ADAS-Cog of ≥3 points. Hypothesis 3 (H3) is as follows: treatment effects will still be detectable 8 weeks postintervention, although not necessarily at 16 and 24 weeks postintervention.

Fourth, estimate the effect of dementia severity on treatment outcomes among patients receiving active rTMS. Hypothesis 4 (H4) is as follows: the effect size will be greater in participants with a clinical dementia rating (CDR) of 1 than in those with a CDR of 2.

### Experimental Design

This is a randomized, double-blind, placebo-controlled clinical trial of rTMS for the treatment of cognitive impairment in patients stratified by severity of Alzheimer dementia. Participants with probable Alzheimer disease will be recruited from the three sites contributing to the study (Winnipeg, Montreal, and Melbourne), and will be randomly assigned to either a 2-week, 4-week, or sham high-frequency rTMS treatment. Standard cognitive assessments will be performed before and after treatment, as well as at scheduled follow-up visits up to 6 months after the end of the intervention. Participants will be blind to the type of treatment (active vs placebo). Assessors will be blind to both the type and duration (2 vs 4 weeks) of treatment.

## Methods

### Recruitment

Recruitment for this study will be performed at all three sites. Patients with probable Alzheimer disease at mild to moderate stages will be recruited. The target recruitment rate of patients at each site is estimated to be approximately 25 per year, with a target total recruitment of 300 participants across all sites.

All potential participants must have been diagnosed with mild or moderate stage Alzheimer disease by their referring physician or one of our study doctors. The screening doctor will complete an eligibility assessment with the potential participants to confirm their suitability to participate in the study. This assessment will use the CDR [49] and MoCA [50] to assess the severity of dementia. In addition, they will complete the Cornell Scale for Depression in Dementia (CSDD) [51] to assess for comorbid depression. The screening doctor will also consider various inclusion and exclusion criteria.

The inclusion criteria are as follows (all must be met): age >55 years; MoCA score between 7 and 25; CDR score of 1 to 2; CSDD score of 18 or less to rule out moderate to severe depression; diagnosis of probable mild or moderate Alzheimer disease as confirmed by the treating neurologist, geriatrician, or psychiatrist, and/or by the study coinvestigators; and use of a stable dose or no dose of an acetylcholinesterase inhibitor for at least 3 months prior to study entry with no plans to change medication for the duration of the study. If a participant decides to discontinue an Alzheimer disease–related medication (ie, a cholinesterase inhibitor), he/she will wait a minimum of 6 weeks prior to the start of the rTMS treatment.

The exclusion criteria are as follows (any of the following): psychiatric conditions/disorders or current neurological or medical disorders, other than Alzheimer disease, that could interfere with cooperative participation (eg, severe agitation and prominent anxiety); diagnosis of intellectual disability; impaired vision or hearing severe enough to impair performance in cognitive tests; exclusive diagnosis of other forms of dementia (including posterior cortical atrophy); primary psychiatric disorders (eg, schizophrenia and bipolar affective disorder) or current and/or unstable neurological, systemic, or medical disorders (eg, liver disease, congestive heart failure, and severe chronic obstructive pulmonary disease [COPD]) that may impair cognition or the ability to complete the required study procedures; use of benzodiazepines and zopiclone during the study and preceding 2 weeks; use of high doses of antipsychotics (based on clinical judgement) that may impair cognition during the study and preceding 2 weeks, or situations where changes in antipsychotic doses can reasonably be anticipated; participation in a clinical trial with any investigational agent within 2 weeks prior to study enrolment; current substance abuse disorder; history of epileptic seizures or epilepsy; contraindication for receiving TMS treatment according to a TMS questionnaire; inability to adequately communicate in English at Manitoba and Australia sites and either English or French at the Montreal site; previous treatment with rTMS within the past 3 months; and any plans to change medication for Alzheimer disease, mood disorders, or pain during the study. Neuropsychiatric symptoms that are considered secondary to Alzheimer disease are not considered in the exclusion criteria, except where they would make it difficult to comply with study requirements, as described above.

Following the initial screening process, we will obtain a magnetic resonance imaging (MRI) scan of the participant’s head. An MRI scan is required by the neuronavigation software in order to position the TMS coil accurately over the target brain region. If a clinical MRI scan already exists, which is suitable for our needs (adequate resolution and coverage to identify and locate internal and external reference points used by the software), we will obtain it from the medical records. If there is no previous clinical MRI scan or the previous MRI scan is not adequate for our research purposes, we will schedule and pay for a research MRI scan to be completed for the participant.

Before performing the MRI scan, we will ask participants if they have any implants, devices, or objects that can be hazardous to them and/or may interfere with the scan. If they express any concerns, we will consult with the MRI clinic and/or ask them to consult with their family doctor. If we still cannot retrieve a valid MRI scan, a reference head model will approximate the participant’s anatomy.

When a scan is performed for the purpose of this study, the following scanning parameters will be used: T1-weighted scan; voxel resolution, 1 mm^3^; matrix, 256×256; and field of view, 25.6 cm (to match the matrix and resolution). The tip of the nose and both ears are required to be included in the scan.

If patients have any medical implants, devices, or objects that could be hazardous to them during the rTMS treatments, we will ask them to consult with their family doctor and provide their doctor’s confirmation via a written document that rTMS can be safely applied for their continuation in the study.

Prior to study participation, all patients and their primary caregivers will be required to sign an informed consent form approved by the ethics board of each site of the study.

### Randomization

Once enrolled in the study, each participant will be assigned to a treatment group (either sham or active, as well as either 2 or 4 weeks of treatment), using stratified block randomization (block size of 3). There will be four distinct stratification blocks using two levels for two factors (age and severity) as follows: age ≥70 years, CDR=1; age ≥70 years, CDR=2; age <70 years, CDR=1; age <70 years, CDR=2.

Group assignment will be determined using an automated algorithm. The only person who will know the group assignment for a given participant is the rTMS administrator at each site who provides the age and CDR data for the algorithm and informs the participant of the duration of treatment. Blinding of the site coordinator will be broken only when necessary to ensure patient safety, that is, in case of a serious adverse event where clinical follow-up is necessary.

### Treatment Protocol

Patients in both the active and sham treatment groups will undergo daily (5 days/week) rTMS treatment. Each treatment will apply 25 trains of rTMS pulses bilaterally to the DLPFC. Each train will have a duration of 1.5 s, and pulses will be applied at 20 Hz (for a total of 30 pulses per train). The intertrain interval will be 10 seconds. Thus, there will be a total of 1500 pulses delivered to the brain per day (25×30=750 per side), resulting in a total of 30,000 pulses applied over the course of 4 weeks of treatment (20 sessions) or 15,000 pulses for 2 weeks of treatment (10 sessions). Each TMS treatment session will take approximately 10 to 25 minutes. Any missed treatment sessions will be made up on the following day, with a minimum 30-minute break between sessions.

The pulses will be applied at 90% to 100% of the RMT of each participant. The RMT, which will be measured for each hemisphere before the first treatment, is determined by applying single TMS pulses over the primary motor cortex and observing the lowest intensity at which it causes an involuntary twitch of the participant’s contralateral thumb. The specific process involves setting the intensity of the stimulator at 65% to 75% of the maximum intensity and adjusting the coil location over the primary motor cortex until the “hotspot” for activating the involuntary twitch is found. Then, the intensity is lowered in 1% decrements to find the lowest intensity at which an involuntary twitch can be clearly observed in three consecutive pulses. The intensity of treatment will be 90% to 100% of the RMT, unless the participant is having trouble tolerating the treatment, in which case treatment at an intensity of 90% of the RMT will be used for the first session and increased to the full dose of 100% by the end of the first week of treatment.

The location of the DLPFC will be determined using the BrainSight 2 navigation system [52] for TMS. The right and left DPLFCs of each participant will be localized using their own MRI scan, and the coil location and direction will be specified using the BrainSight software at Talairach coordinates (x, y, z) = (–50, 30, 36). The coil will be held at approximately 45 degrees relative to the horizontal axis, but this will be measured approximately rather than specified exactly using the neuronavigation software.

To prevent unblinding, a Magstim sham coil will be used for sham treatments. This coil provides the same sound and tactile sensory experience as the real coil, but it attenuates the strength of the induced electrical field in the brain well below the threshold required to stimulate neurons. In addition, during treatment, only the participant and the research personnel designated to administer the rTMS will be present (no caregiver).

### Outcome Measures

Each participant will attend six assessment days in total as follows: baseline (week 0) and week 3, week 5, week 8, week 16, and week 24 posttreatment sessions. At each assessment day, three assessments will be given to the participant in the following fixed sequence: ADAS-Cog, verbal fluency test, and Stroop test. Only the patient and the research personnel designated to administer assessments will be in the room during assessments. At the baseline and week 5 and week 16 posttreatment assessments, additional caregiver assessments will be performed. Alzheimer Disease Co-operative Study-Activities of Daily Living Inventory (ADCS-ADL) and Neuropsychiatric Inventory Questionnaire (NPI-Q) assessments will be performed at each of these visits (in that order), while the Treatment Satisfaction Questionnaire for Medication (TSQM) will only be used at the week 5 assessment. These outcome measures must be administered to the same caregiver at each visit. If a participant is accompanied to an assessment visit by someone other than their usual caregiver, the assessor will contact the usual caregiver by phone to complete these assessments.

All assessments will be performed on a Monday or the first working day of the week. Note that the specific dates of the last three assessments (the follow-up assessments) will be adjusted based on whether the participant is in a 2-week or 4-week treatment group. An assessor will be assigned to each participant. That same assessor must perform all of the six assessments with the assigned participant. Deviations should be justified (changes in staff, assessor illness, etc), and an explanation will be documented in the assessment notes.

As participant schedules are often busy, we can adjust the specific dates of the assessments within ranges (Table 1). If no assessment is possible within these windows, the assessment will be skipped and the data point will be missing from the analysis (with the exception of the baseline assessment, which will require rescheduling the treatment).

The primary outcome measure will be the change in patient scores from baseline on the ADAS-Cog assessment, as that is the most common standard test used in dementia clinical trials. Alternate forms of the ADAS-Cog word lists will be used at each visit to avoid possible practice effects. The secondary outcome measures will be the change in the scores from baseline of the proximal measures Stroop test [53] and verbal fluency test, as well as the distal measures NPI-Q and ADCS-ADL. We will also assess the tolerability of the rTMS treatment by the TSQM [54], which will be completed by patients and their primary caregivers posttreatment.

At each site, the above assessments will be administered by a study research assistant (RA) blinded to the group assignment of the patients. The RAs involved in administering treatment or assessments will not be involved in any of the statistical data analyses.

**Table 1:** Date adjustments for assessments.

Assessment	Range of possible dates
Baseline	Up to 1 week before the first treatment
Week 3	+/− 1 day of the ideal date
Week 5	+/− 1 week of the ideal date, but after the final treatment
Week 8	+/− 1 week of the ideal date
Week 16	+/− 2 weeks of the ideal date
Week 24	+/− 2 weeks of the ideal date

### Safety Considerations

Before enrolling in the study, all participants will be screened for possible rTMS contraindications (seizure history, brain lesions, metallic implants, etc). Participants will be asked at each visit if they have experienced any adverse effects from the treatment. Any reported adverse events or effects will be recorded, and treatment will be suspended at the discretion of the study physicians if the adverse effect is considered to be serious (ie, life threatening or requiring hospitalization or medical treatment). The participant’s self-assessment of any pain or discomfort from the treatment will also be recorded at every visit.

A data and safety monitor board (DSMB) will oversee the study to ensure that proper safety procedures are followed and that adverse effects of the treatment are properly documented. If adverse effects are discovered that warrant a review or investigation before proceeding with the study, the DSMB will have the authority to initiate such a review. The DSMB will also ensure that the study does not deviate from the intended protocol.

### Ethical Considerations and Follow-Up

Following the 24-week posttreatment assessment, participants will be informed of their assigned treatment group. Patients randomized to the sham treatment will be offered 2 weeks or 4 weeks of active treatment. The patients and/or their family can choose the duration of treatment. Any participant who experiences an adverse effect will be followed up by a study physician until the adverse effect has resolved.

### Data Management

Study data (participant medical and demographic data, treatment records, and assessment results) will be maintained on a password-protected database accessible only to active research team members, with access to specific information, such as group assignment, and assessment results restricted based on the individual’s assigned role in the study. Regular backups of the database will be performed and stored on a secure server. Identifying information (name, phone number, address, etc) will not be stored on the same system as the study data and will only be accessible to staff members who need to contact participants.

### Statistical Analysis

#### Sample Size Estimation

The sample size for the hypothesis that one group’s mean is statistically significantly different from the other (assuming two equally sized groups, which is the case in this study) can be calculated using the following formula [55]:


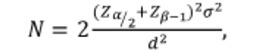


where *Z_α_* is the upper tail critical value in the standard normal distribution at the α level of significance (it is 1.96 and 2.58 for 5% and 1% significance levels, respectively), *Z_β-1_* is the normal deviate at 1−β% power with β% of type II error (it is 0.84 and 1.28 for 80% and 90% power, respectively), and *σ* and *d* are the standard deviation and difference of means of the two groups, respectively. The calculated N is the number of subjects in each group.

Using the above formula with a significance level of .05 (corrected using the Bonferroni adjustment for three treatment groups), an expected difference of 3 points on the ADAS-Cog score (derived from a study by Rabey et al [7], which also used ADAS-Cog as a primary outcome measure), a standard deviation of 4.9 points (derived from the results of our own pilot study [8]), and a power level of 80%, the minimum sample size is estimated to be 63 participants per group (189 in total). Allowing for 10% drop out, we will need to enroll at least 208 patients in the study to achieve this power.

#### Analysis of the Results

Baseline characteristics will be assessed between the active and sham treatment groups by descriptive statistics, as well as formal statistical tests. Baseline differences between the two groups, if any, will be adjusted for in the final statistical analyses. All assessment scores will be checked for normality of distribution to determine the choice of either parametric or nonparametric methods. Bartlett statistic will be used to assess the homogeneity of variances, and Levene [56] or Brown-Forsythe [57] tests, which are less sensitive to departures from normality, may also be used. In all instances, a *P* value <.05 will be considered significant.

To test H1 and H2 (the efficacy of rTMS treatment), two-factor (active vs sham) repeated (pre-post treatment) analysis of variance (ANOVA) will be used to investigate the effect of treatment before and after the 4-week block of treatment. The dependent variable will be the change in the ADAS-Cog score (primary outcome measure). Testing H3 will be similar to the tests of H1 and H2, but the two factors will be the two durations of treatment.

For secondary outcome measures, a two-factor ANOVA will also be used in the same way as described above, but a Hochberg test [58] will be applied to correct for multiple comparisons.

The last-observation-carried-forward method will be used in the case of missing data or premature termination.

To test H4 (the durability of rTMS treatment), repeated measure ANOVA will be used among responders (those who show improvement in the ADAS-Cog score of ≥3 points over the course of treatment [3] in both groups) to investigate the duration over which the improvement may last. Post-hoc follow-up methods, such as Dunnett [59] and Tukey [60] tests, will be applied as needed. In addition, a mixed regression model will be developed to predict the response variable at each assessment visit of the active treatment group (both treatment arms separately). The independent variables will be patients’ current age and the severity of Alzheimer disease (as measured by the CDR).

To test H5 (the correlation of treatment effect and severity), we will run regression and correlation analyses between the severity of Alzheimer disease (the MoCA and CDR scores) at baseline and the change in the ADAS-Cog score. The correlation coefficient (either the Pearson or Spearman correlation coefficient, whichever is more appropriate) and its statistical significance will be determined. Sensitivity of the regression and correlation analyses with respect to the data distribution will be assessed. Influential observations and outliers, if any, will be identified.

The above statistical analysis will also be repeated for secondary outcome measures after correcting for multiple comparisons. In addition, covariate analysis will be performed in an appropriate manner. Covariance balance (or imbalance) is checked with the ANOVA test when there are three or more groups or by the *t* (or the approximate Z) test when there are only two groups. For the ANOVA test, we will first check the satisfaction of ANOVA assumptions (such as normality of distribution and equality of variances). If any violation is found, transformations will be made before the ANOVA test. If covariance imbalance is statistically significant, the final data analysis will be adjusted for covariance imbalance (eg, by the use of regression analysis with these imbalanced covariates as explanatory variables).

### Quality Assurance

A designated study coordinator will travel and visit the study sites approximately three times per year to ensure that each site is adhering to the study protocol. They will also ensure that the three sites are consistent in the details of implementing the study protocol, such as RMT measurement and assessment techniques.

Before the study starts, after the RAs are hired, the principal investigator (PI) and co-PIs at each site will have an online meeting to go through the protocol and procedure entirely. All the RAs will be trained and will fully practice their specific tasks before the study starts. If there is any issue of ambiguity raised during this meeting, the PI and co-PIs of the sites may schedule a follow-up meeting to discuss the issue and ensure the group is in agreement on the intended protocol.

The study coordinator’s job is to ensure that the three sites are synchronized in terms of adherence to the protocol. Before the first visit of the site coordinator, the PIs of each site will meet virtually by video conferencing to go over the details of the protocol and how they plan to ensure the staff at each site will be trained to perform the protocol in exactly the same manner at every site. The first visit of the coordinator will be during the first months of the study when the treatment of the first block of patients starts. During each visit, the study coordinator will attend at least one treatment and one assessment to be able to evaluate the procedures and flag any inconsistencies across the sites. In addition, the coordinator is responsible to oversee the overall data management in the main server located in Manitoba, as well as monitor and communicate across the three sites on an ongoing basis to ensure compliance with the protocol at every site.

All outcome assessment data will be uploaded and saved on the PI’s Biomedical Engineering (BME) server in Manitoba, which is a secure server that is being administered by a dedicated computer engineer. A standard routine of anonymization will be in place to assign a code to each patient and ensure the safety and security of patient data.

A process of cross-validating data entry will be established between the three sites. All assessment scores will be recorded in the study database along with scanned copies of the supporting assessment documents. Assessors will check the scoring and data entry performed by other assessors (Winnipeg assessments will be checked by Melbourne, Melbourne assessments will be checked by Montreal, and Montreal assessments will be checked by Winnipeg). The purpose of this is to ensure data quality by catching and correcting errors, as well as requiring site assessors to communicate regularly about assessment scoring guidelines.

### Ethics

All participants (or their caregivers in cases where the caregivers are legal representatives acting on behalf of the participants) will read and sign an informed consent form before being enrolled in the study. A trained RA will discuss the study and the consent form with the participants and their caregivers prior to signing and answer any questions they may have. Participants may withdraw from the study at any time without being required to offer an explanation.

The consent forms and the overall study protocol must be approved by the local ethics board for each site of the study prior to the commencement of the study.

### Plausible Side Effects

There are some known and expected side effects of rTMS treatment, which will be mentioned in the consent forms to inform participants of the study. The most common side effects of rTMS are as follows: (1) headache (usually mild) following rTMS application that is believed to be due to muscle tension and (2) toothache and pain in the eye, scalp, and neck that are all reported to be mild and temporary following rTMS application. The more serious side effect of rTMS is the risk of seizures in people with a history of epilepsy or in people who have an increased risk of seizures. For this reason, we will screen carefully to exclude those with a history of seizures or increased risk of seizures.

### Interim Analysis and Plausible Changes to the Protocol

We will run an interim analysis after enrollment of 150 participants and after at least 100 of them have finished at least 16 weeks of the study. Through the interim analysis, we will try to answer the question of whether the trial is likely to reach its objective if continued to the planned maximum sample size and/or whether the treatment protocol should be modified, for example, in case a disease-modifying therapy is discovered in the course of the study. In case of the latter, the team will write an amendment to the funding agency and then, upon their approval, will submit the amendment to the ethics board for the modified protocol and will inform the participants.

To ensure the double-blind nature of the study is not compromised by the interim analysis, all eligible data to be included in the analysis will be randomly assigned as P1, P2, P3, etc by the PI. Only the PI and one main coordinator will have access to the master file that associates the P1, P2, etc labels to the codes of the study subjects. The data will be analyzed by a RA blinded to that data under the supervision of our collaborator statistician (XW), who will also remain blind to the group identity within the data. The arms of the intervention will be named randomly as G1, G2, G3, and G4 by the PI to avoid any bias.

## Results

### Recruitment and Enrollment

As of November 1, 2020, we have screened 523 individuals, out of which 133 were eligible and have been enrolled. Out of the 133 individuals enrolled and randomized to the intervention groups, 104 have completed the study and 20 have discontinued the study or have withdrawn for various reasons at various stages of the study. Data of some withdrawn individuals who completed the study up to week 8 or 16 are still usable for analysis. Three individuals withdrew because they found the treatment uncomfortable, nine withdrew with no reason given, and eight were withdrawn by the site PI. Out of the eight withdrawn by the site PI, two were withdrawn for safety reasons as they developed some illnesses, although they were not related to the rTMS treatment, four were noncompliant (changed their medication during the study) or found the rTMS pulses unbearable, and two could not finish the treatment due to pandemic lockdown.

Recruitment and enrollment have been slower than initially anticipated, and the pandemic has slowed these even further. Before the pandemic, the realistic recruitment rate was one per month.

### Adverse Events

We have developed a series of detailed questions to mark any plausible adverse effect of the treatment, and the series is used consistently among the different sites of the study. As of November 1, 2020, there has been no serious adverse event. However, 89 of the participants reported expected adverse events as described in the Plausible Side Effects section above. Twelve participants reported some unexpected adverse events, which were most likely unrelated to the rTMS treatment. The reported events were increased blood pressure on one day, a nightmare, vivid dreaming, sleeping trouble, disorientation, blurry vision, and unsteadiness on the feet for a few minutes. All reported cases were temporary and reported on only one day. All issues resolved without medication. All adverse events have been described in detail in quarterly DSMB reports.

### Minor Deviations From the Treatment Protocol

For those participants finding rTMS pulses painful, we will administer pulses lower than 90% to 100% of the RMT threshold in the first three sessions of treatment and slowly increase the value to 90% of the RMT for the rest of the sessions.

## Discussion

Overall, the study has been continuing as expected. In general, participants have found the rTMS treatment tolerable and have been compliant to the study protocol. One interesting fact is that we found our participants eager to continue the study even during the pandemic. However, the study has become slow because of the lockdowns imposed by the universities and health authorities.

## Appendix

Multimedia Appendix 1 Funding agency reviews of the proposal.

## Abbreviations

ADAS-Cog: Alzheimer Disease Assessment Scale-Cognitive Subscale
ADCS-ADL: Alzheimer Disease Co-operative Study-Activities of Daily Living Inventory
ANOVA: analysis of variance
BDNF: brain-derived neurotrophic factor
CDR: clinical dementia rating
CSDD: Cornell Scale for Depression in Dementia
DLPFC: dorsolateral prefrontal cortex
DSMB: data and safety monitor board
GABA: gamma aminobutyric acid
Glx: glutamate-glutamine
MoCA: Montreal Cognitive Assessment
MRI: magnetic resonance imaging
NPI-Q: Neuropsychiatric Inventory Questionnaire
PI: principal investigator
RA: research assistant
RMT: resting motor threshold
rTMS: repetitive transcranial magnetic stimulation
TMS: transcranial magnetic stimulation
TSQM: Treatment Satisfaction Questionnaire for Medication
